# Surveys of Forest Birds on Puerto Rico, 2015

**DOI:** 10.3897/BDJ.5.e20745

**Published:** 2017-11-22

**Authors:** John D Lloyd, Christopher C Rimmer

**Affiliations:** 1 Vermont Center for Ecostudies, White River Junction, United States of America

**Keywords:** Puerto Rico, Bicknell's Thrush, Catharus bicknelli, point counts, birds, Elfin-woods Warbler, Setophaga angelae

## Abstract

**Background:**

The island of Puerto Rico supports a diverse assemblage of breeding birds, including 16 endemic species (Raffaele et al. 1998), and provides critical wintering habitat for many North American migratory birds (Wunderle and Waide 1994). Despite being a hotspot of avian biodiversity, spatially extensive data on the distribution and abundance of birds on the island are scarce. Breeding-bird assemblages were sampled by the North American Breeding Bird Survey from 1997–2007 (Sauer et al. 2013), but comparable primary data are not available for bird assemblages present during the boreal winter.

**New information:**

We provide data from one of the few spatially extensive surveys of forest birds on Puerto Rico. We sampled 211 locations in forests across the island during January–March 2015 using repeated point-count surveys. These data are suitable for use in estimating abundance, occupancy, and distribution of forest birds on Puerto Rico during the winter.

## Introduction

Puerto Rico, the easternmost island of the Greater Antilles, is a hotspot of biodiversity (Myers et al. 2000). It supports an especially rich avifauna; 354 bird species are known from the island (Mendez-Gallardo and Salgeuro-Faria 2008) including 16 endemic species (Raffaele et al. 1998) and, during the boreal winter (hereafter, winter), many migratory species that arrive from North America (Wunderle and Waide 1994, Wunderle and Waide 1993). Although general patterns of distribution are well-described for many bird species inhabiting the island, few spatially extensive data sets exist that are suitable for estimating important population characteristics such as abundance or occupancy rate. Breeding-bird assemblages on Puerto Rico were sampled by the North American Breeding Bird Survey from 1997–2007 (Sauer et al. 2013), yet comparable data from the non-breeding season do not exist. Low-granularity summaries of bird distribution and relative abundance across the island during winter exist (Wunderle and Waide 1993), as do numerous site-specific studies of bird assemblages during winter (e.g., Faaborg et al. 2007), but primary data from replicated, spatially extensive studies are lacking.

Here, we provide data from repeated point-count surveys conducted at 211 locations in forests across the island during January–March 2015. At each point, an observer counducted 4 sequential surveys, each lasting 2.5 minutes, during which all birds heard or seen were recorded. In addition, the observer estimated the distance to each individual bird that was detected. The structure of these data makes them amenable to a variety of analytical approaches, including occupancy models, N-mixture models, and distance sampling (MacKenzie et al. 2002, Royle 2004, Buckland et al. 2005). An additional advantage from the standpoint of statistical inference is that, within the confines of the sampling frame, which was based on the potential distribution of Bicknell's Thrush (*Catharus bicknelli*) (McFarland et al. 2013), the points were located using a spatially balanced, randomized design. As such, these data may prove useful for estimating spatial variation in species-specific parameters, such as abundance, as well as in community metrics, such as species richness.

## General description

### Purpose

The overall goal of this research was to describe the distribution and abundance of forest birds on Puerto Rico during the winter by conducting repeated point-count surveys of birds at forested locations across the island. The focal species for these surveys was Bicknell's Thrush (*Catharus bicknelli*) and thus surveys were concentrated in areas considered potentially suitable for this species. As such most, but not all, survey locations were in submontane evergreen forest or lower montane elfin, sierra palm, transitional, and tall cloud forest (Kennaway and Helmer 2007).

## Project description

### Title

Surveys of Forest Birds on Puerto Rico, 2015

### Personnel

Christopher C. Rimmer, John D. Lloyd, and Jose Salguero Faria

### Study area description

The study area consisted of 211 locations on Puerto Rico where we conducted point-count surveys for birds during January–March 2015. Survey points were located by first drawing a generalized random tessellation stratified (GRTS) sample of 1-km^2^ cells that were identified from the Bicknell’s Thrush winter-habitat model (McFarland et al. 2013). We then visited each cell and identified 3-5 locations suitable for point-count surveys. Suitability was based on extent of forest cover – at least 50% of the area within 50 m of the point was forested – and accessibility; all points were along public roads or trails. To maintain independence of counts conducted at different points, we placed each point at least 250 m from its nearest neighbor. We were able to establish survey points and conduct surveys in 43 different cells. Most points were in submontane evergreen forest or lower montane elfin, sierra palm, transitional, and tall cloud forest (Kennaway and Helmer 2007). The elevation of survey points across all cells ranged from 0–1,297 meters above sea level (MASL), with a median of 705 MASL.

### Funding

National Fish and Wildlife Foundation grant no. 48324 and private donations from friends of the Vermont Center for Ecostudies

## Sampling methods

### Study extent

Surveys were conducted in forested areas at a variety of elevations on Puerto Rico.

### Sampling description

We conducted standardized point-count surveys for birds at 211 locations on Puerto Rico during January – March 2015. Survey points were located by first drawing a GRTS sample of 60 1-km^2^ cells that were identified from a predictive model of Bicknell’s Thrush winter habitat (McFarland et al. 2013). The sample included an unequal mix of cells with a high (>0.5; n = 30), medium (0.25–0.50; n = 20), and low (<0.25; n = 10) predicted probability of occurrence of Bicknell’s Thrush. Once we had drawn a sample of cells to survey, we visited each cell and identified 3-5 locations suitable for point-count surveys. Suitability was based on the extent of forest cover – at least 50% of the area in a 50-m radius around each point was forested – and accessibility; all points were along public roads or trails. To maintain independence of counts conducted at different points, we placed each point at least 250 m from its nearest neighbor. Survey points were georeferenced via GPS. Of the 60 selected cells, we were able to visit and survey 43 during 2015 Fig. 1. Most (n = 29) of the cells that we sampled had a high predicted probability of occurrence of Bicknell’s Thrush; relatively fewer of the sampled cells had a medium (n = 5) or low (n = 9) predicted probability of occurrence. Survey points within the cells covered a wide range of elevations: from 0–1,297 meters above sea level (MASL), with a median of 705 MASL. Exact geographic coordinates of each survey point are contained in the data package available at KNB (see link below in section 'Data resources').

At each survey point, an observer conducted 4, 2.5-minute counts during which all birds heard or seen were recorded (i.e., a repeated-counts design). Counts were conducted in sequence, one after the other. A 1-minute playback of Bicknell's Thrush vocalizations preceded the second and fourth counts. Individuals of all species detected during each count were recorded into four distance bands: 0–10 m from the observer, 10–25 m, 25–50 m, and >50 m from the observer. All surveys were conducted between sunrise and 10:00 Atlantic Standard Time. Surveys were not conducted during rain or high winds.

### Step description

Step 1. Identification of sampling locations.

We downloaded the raster output of the winter habitat model of Bicknell's Thrush (McFarland et al. 2013) from Data Basin (http://databasin.org). Each 1-km^2^ cell in the raster included an associated predicted probability of occurrence of suitable habitat. We selected 60 cells in which to conduct point-count surveys using a GRTS sampling design. The sample included 30 cells with a high (>0.50) probability of containing suitable habitat, 20 with a medium (0.25–0.50) probability, and 10 with a low (<0.25) probability.

Step 2. Establishing sampling points.

We visited 43 of the selected cells during 2015 (due to the paucity of detections of the focal species, Bicknell's Thrush, we did not return as planned in 2016 to sample the remaining cells). In each cell, we identified 3–5 locations suitable for conducting point-count surveys. Point locations within each cell were chosen systematically based on ease of access - all points were located in forests adjacent to public roads or trails - and so as to cover as much of the cell as possible. Each point was also placed so that forest cover within a 50-m radius was at least 50%. We located survey points >250 m from one another so as to maintain the independence of counts at each point. The location of each point was established in the field using a handheld GPS unit. Coordinates were not properly recorded for 28 points and so the exact the location of the survey point is unknown.

Step 3. Conduct bird surveys.

At each survey point, an observer conducted 4, 2.5-minute counts during which all birds heard or seen were recorded (i.e., a repeated-counts design). Counts were conducted in sequence, one after the other. A 1-minute playback of Bicknell's Thrush vocalizations preceded the second and fourth counts. Individuals of all species detected during each count were recorded into four distance bands: 0–10 m from the observer, 10–25 m, 25–50 m, and >50 m from the observer.

## Geographic coverage

### Description

Puerto Rico

### Coordinates

17.375 and 19.125 Latitude; -67.875 and -65.0 Longitude.

## Taxonomic coverage

### Taxa included

**Table:** 

Rank	Scientific Name	
species	*Accipiter striatus*	
species	*Anthracothorax dominicus*	
species	*Anthracothorax viridis*	
species	*Ardea alba*	
species	*Brotogeris versicolurus*	
species	*Bubulcus ibis*	
species	*Buteo jamaicensis*	
species	*Butorides virescens*	
species	*Cathartes aura*	
species	*Catharus bicknelli*	
species	*Chlorostilbon maugaeus*	
species	*Coccyzus minor*	
species	*Coccyzus vieilloti*	
species	*Coereba flaveola*	
species	*Columba livia*	
species	*Columbina passerina*	
species	*Contopus latirostris*	
species	*Crotophaga ani*	
species	*Cypseloides niger*	
species	*Elaenia martinica*	
species	*Estrilda melpoda*	
species	*Eulampis holosericeus*	
species	*Euphonia musica*	
species	*Eupsittula canicularis*	
species	*Falco sparverius*	
species	*Geotrygon chrysia*	
species	*Geotrygon montana*	
species	*Icterus icterus*	
species	*Icterus portoricensis*	
species	*Loxigilla portoricensis*	
species	*Margarops fuscatus*	
species	*Megascops nudipes*	
species	*Melanerpes portoricensis*	
species	*Mimus polyglottos*	
species	*Mniotilta varia*	
species	*Molothrus bonariensis*	
species	*Myiarchus antillarum*	
species	*Nesospingus speculiferus*	
species	*Orthorhyncus cristatus*	
species	*Parkesia motacilla*	
species	*Passer domesticus*	
species	*Patagioenas leucocephala*	
species	*Patagioenas squamosa*	
species	*Petrochelidon fulva*	
species	*Progne dominicensis*	
species	*Quiscalus niger*	
species	*Seiurus aurocapilla*	
species	*Setophaga adelaidae*	
species	*Setophaga americana*	
species	*Setophaga angelae*	
species	*Setophaga caerulescens*	
species	*Setophaga citrina*	
species	*Setophaga discolor*	
species	*Setophaga magnolia*	
species	*Setophaga ruticilla*	
species	*Setophaga tigrina*	
species	*Setophaga virens*	
species	*Spermestes cucullata*	
species	*Spindalis portoricensis*	
species	*Thalasseus maximus*	
species	*Tiaris bicolor*	
species	*Tiaris olivaceus*	
species	*Todus mexicanus*	
species	*Turdus plumbeus*	
species	*Tyrannus caudifasciatus*	
species	*Tyrannus dominicensis*	
species	*Vireo altiloquus*	
species	*Vireo latimeri*	
species	*Zenaida asiatica*	
species	*Zenaida aurita*	

## Temporal coverage

### Notes

2015-01-22 through 2015-03-30

## Usage rights

### Use license

Creative Commons Public Domain Waiver (CC-Zero)

### IP rights notes

This dataset is released to the public and may be freely downloaded. Please keep the designated Contact person informed of any plans to use the dataset. Consultation or collaboration with the original investigators is strongly encouraged. Publications and data products that make use of the dataset must include proper acknowledgment.

## Data resources

### Data package title

Surveys of Forest Birds on Puerto Rico, 2015

### Resource link

https://knb.ecoinformatics.org/#view/doi: 10.5063/F1MG7MKK

### Alternative identifiers

doi: 10.5063/F1MG7MKK

### Number of data sets

2

### Data set 1.

#### Data set name

associatedOccurrences.csv

#### Data format

Darwin Core

#### Number of columns

12

#### Download URL

https://knb.ecoinformatics.org/knb/d1/mn/v2/object/jlloyd.13.14

#### Description

A data table containing information on species occurrences generated during sampling events conducted January - March 2015 on Puerto Rico. This data table is linked to the resource samplingEvents.csv by the eventID.

**Data set 1.** 

Column label	Column description
parentEventID	Alphanumeric identifer that identifies survey points within a locality. Of the format: Site identifier-point identifier.
eventID	Alphanumeric identifier for each survey occasion. Of the format: Site identifier-point identifier.survey identifier.
occurrenceID	Alphanumeric identifer for the occurences during a survey event, of the format: Site identifier-point identifier.survey identifier-occurrence identifier. Occurrences consist of detections of individual birds or groups of birds.
individualCount	The number of individuals represented present at the time of the occurrence.
organismQuantity	A number value for the quantity of organisms. Same as individualCount.
organismQuantityType	The type of quantification system used for the quantity of organisms.
scientificName	The full scientific name of the organism associated with the occurrence.
kingdom	The full scientific name of the Kingdom of the organism associated with the occurrence.
phylum	The full scientific name of the phylum or division of the organism associated with the occurrence.
class	The full scientific name of the class of the organism associated with the occurrence.
taxonRank	The taxonomic rank of the most specific name in the scientificName.
dynamicProperties	Additional characteristics about the record, indicating the distance in meters between the observer and the individual at the time of detection.

### Data set 2.

#### Data set name

samplingEvents.csv

#### Data format

Darwin Core

#### Number of columns

14

#### Download URL

https://knb.ecoinformatics.org/knb/d1/mn/v2/object/jlloyd.15.1

#### Description

A data table containing information on sampling events conducted January - March 2015 on Puerto Rico. This data table is linked to the resource associatedOccurences.csv by the eventID.

**Data set 2.** 

Column label	Column description
parentEventID	Alphanumeric identifer that identifies survey points within a locality. Of the format: Site identifier-point identifier.
eventID	Alphanumeric identifier for each survey occasion. Of the format: Site identifier-point identifier.survey identifier.
samplingProtocol	The name of, reference to, or description of the method or protocol used during an Event.
sampleSizeValue	A numeric value for a measurement of the size (time duration, length, area, or volume) of a sample in a sampling event.
sampleSizeUnit	The unit of measurement of the size (time duration, length, area, or volume) of a sample in a sampling event.
eventDate	The date-time or interval during which an Event occurred.
eventTime	The time or interval during which an Event occurred, with a format of hh:mm-UTC offset[hh:mm]
country	The name of the country or major administrative unit in which the Location occurs.
countryCode	The standard code for the country in which the Location occurs. Codes here are ISO 3166-1-alpha-2 country codes.
locality	The specific description of the place where the sampling event occurred.
decimalLatitude	The geographic latitude (in decimal degrees, using the spatial reference system given in geodeticDatum) of the geographic center of a Location. Positive values are north of the Equator, negative values are south of it. Legal values lie between -90 and 90, inclusive.
decimalLongitude	The geographic longitude (in decimal degrees, using the spatial reference system given in geodeticDatum) of the geographic center of a Location. Positive values are east of the Greenwich Meridian, negative values are west of it. Legal values lie between -180 and 180, inclusive.
geodeticDatum	The ellipsoid, geodetic datum, or spatial reference system (SRS) upon which the geographic coordinates given in decimalLatitude and decimalLongitude as based.
dynamicProperties	Additional characteristics about the record, indicating whether or not a recorded playback of Bicknell's Thrush vocalizations was broadcast prior to the sampling event

## Figures and Tables

**Figure 1.**
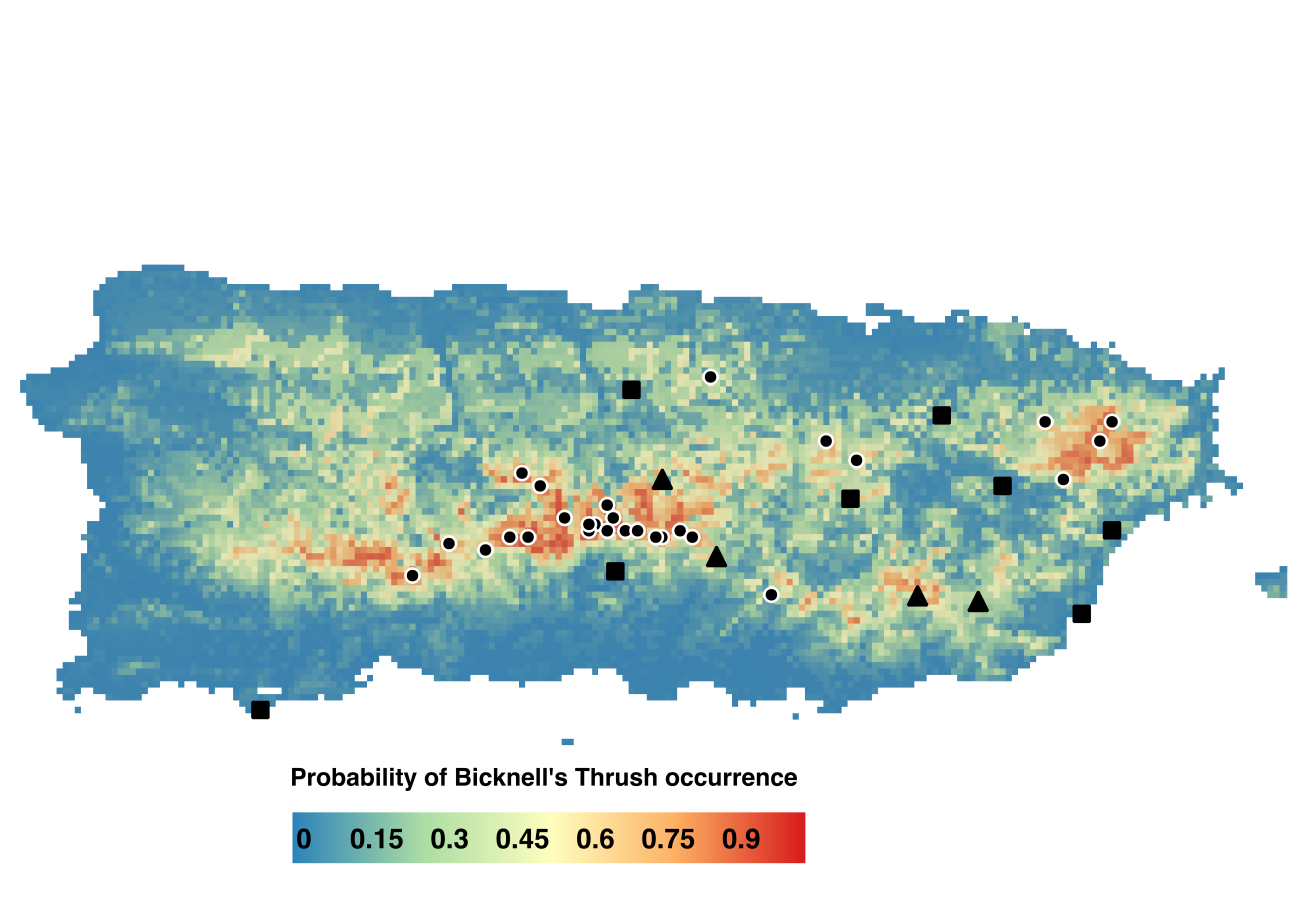
Location of 1-km2 cells on Puerto Rico surveyed for forest birds during 2015. Geographic distribution of sampling locations was based on the predicted probability of occurence of Bicknell's Thrush: 29 cells had a high predicted probability of occurrence of Bicknell’s Thrush (black dots); 5 had a medium (black triangles) and 9 had a low (black squares) predicted probability of occurrence (note that GPS coordinates were not recorded for 1 cell in each category, so only the 40 cells with coordinates are shown).
